# EVALUATION OF QUALITY INDICATORS OF SCREENING COLONOSCOPY PERFORMED IN A PRIVATE QUARTERNARY HOSPITAL IN BRAZIL

**DOI:** 10.1590/0102-6720202400022e1815

**Published:** 2024-08-12

**Authors:** Diogo Turiani Hourneaux de MOURA, Luiza Martins BARONI, Alexandre Moraes BESTETTI, Mateus Pereira FUNARI, Rodrigo Silva de Padua ROCHA, Marcos Eduardo Lera dos SANTOS, Saullo Queiroz SILVEIRA, Eduardo Guimarães Hourneaux de MOURA

**Affiliations:** 1 Instituto D´Or de Pesquisa e Ensino, Hospital Vila Nova Star, Gastrointestinal Endoscopy Division – São Paulo (SP), Brazil.; 2 Universidade de São Paulo, Faculty of Medicine, Gastrointestinal Endoscopy Unit, Department of Gastroenterology – São Paulo (SP), Brazil.; 3 Instituto D´Or de Pesquisa e Ensino, Hospital Vila Nova Star, Anestesiology Department – São Paulo (SP), Brazil.

**Keywords:** Colorectal Neoplasms, Adenoma, Colonoscopy, Neoplasias Colorretais, Adenoma, Colonoscopia

## Abstract

**BACKGROUND:**

Colorectal cancer is the third most common type of cancer in Brazil, despite the availability of screening methods that reduce its risk. Colonoscopy is the only screening method that also allows therapeutic procedures. The proper screening through colonoscopy is linked to the quality of the exam, which can be evaluated according to quality criteria recommended by various institutions. Among the factors, the most used is the Adenoma Detection Rate, which should be at least 25% for general population.

**AIMS:**

To evaluate the quality of the screening colonoscopies performed in a quarternary private Brazilian hospital.

**METHODS:**

This is a retrospective study evaluating the quality indicators of colonoscopies performed at a private center since its inauguration. Only asymptomatic patients aged over 45 years who underwent screening colonoscopy were included. The primary outcome was the Adenoma Detection Rate, and secondary outcomes included polyps detection rate and safety profile. Subanalyses evaluated the correlation of endoscopic findings with gender and age and the evolution of detection rates over the years.

**RESULTS:**

A total of 2,144 patients were include with a mean age of 60.54 years-old. Polyps were diagnosed in 68.6% of the procedures. Adenoma detection rate was 46.8%, with an increasing rate over the years, mainly in males. A low rate of adverse events was reported in 0.23% of the cases, with no need for surgical intervention and no deaths.

**CONCLUSIONS:**

This study shows that high quality screening colonoscopy is possible when performed by experienced endoscopists and trained nurses, under an adequate infrastructure.

## RESUMO

**RACIONAL:**

O câncer colorretal é o terceiro tipo de câncer mais comum no Brasil apesar dos métodos de rastreamento que reduzem seu risco. A colonoscopia é o único método com possibilidade terapêutica. O adequado rastreio por colonoscopia está relacionado à qualidade dos exames, avaliados de acordo com critérios de qualidade recomendados por diversas instituições. Dentre os fatores, o mais utilizado é a Taxa de Detecção de Adenoma, que deve ser de pelo menos 25% na população geral.

**OBJETIVOS:**

Avaliar a qualidade das colonoscopias de rastreamento do câncer colorretal realizadas em um centro brasileiro.

**MÉTODOS:**

Estudo retrospectivo avaliando os indicadores de qualidade em colonoscopias de rastreio nos pacientes assintomáticos com mais de 45 anos. O desfecho primário foi a Taxa de Detecção de Adenoma, e os secundários incluíram a taxa de detecção de pólipos e a segurança do procedimento. Subanálises incluíram a correlação dos achados endoscópicos com sexo e idade e a evolução das taxas de detecção de adenoma ao longo dos anos.

**RESULTADOS:**

Foram incluídos 2.144 pacientes, com media de idade de 60.54 anos. Pólipos foram diagnosticados em 68,6% dos procedimentos. A taxa de detecção de adenoma foi de 46,8%, aumentando ao longo dos anos, principalmente em homens. A taxa de efeitos adversos foi de 0.23%, sem necessidade de intervenção cirúrgica ou registro de óbitos.

**CONCLUSÕES:**

Este estudo demonstra que é possível realizar colonoscopias com qualidade para o rastreio do câncer colorretal quando efetuadas por médicos experientes e enfermeiras treinadas em hospitais com infraestrutura adequada.

## INTRODUCTION

Colorectal cancer (CRC) is the third most common type of cancer in Brazil (excluding non-melanoma skin tumors), with a higher incidence in the Southeast region. The estimated risk of CRC in Brazil per year between 2023 and 2025 is 21.10 cases per 100,000 inhabitants, resulting in a total of 45,630 cases^9^.

CRC is considered a preventable cancer, and there is strong evidence that screening reduces its risk^3,28^. Therefore, screening is recommended for patients over 45 years old (average-risk adults), using tests such as fecal occult blood testing, fecal immunochemical testing (FIT), multitarget stool DNA-FIT, sigmoidoscopy, computed tomographic colonography, and colonoscopy^18,20,33,35^. Recently, in Brazil, aiming to inform the population and increase the adhesion to the CRC screening program, the Chamber of Deputies approved the law (Bill 5024/2019) that establishes March as the month of CRC prevention awareness^20,31^. Colonoscopy is considered the gold standard method, and a high quality procedure is key to avoid undesired outcomes^32^. Quality indicators include preprocedure, intraprocedure, and post-procedure factors^31,34^. The adenoma detection rate (ADR) has been validated as a strong predictor of CRC risk after colonoscopy, and is now the most used quality measure. Since several measures can enhance the ADR, variability in its levels is often reported^19,21,37,39^.

In Brazil, there is a lack of a cross-sectional studies^11,22^ presenting quality indicators such as ADR. This fact may be attributed to a deficient infrastructure in most centers, leading to unsatisfactory results. Therefore, the aim of this study is to evaluate the outcomes of a Brazilian center when adequate infrastructure is provided.

## METHODS

### Study design

This is a retrospective study analyzing the quality indicators of screening colonoscopies performed in a Quaternary Private Hospital, named Vila Nova Star — Rede D’Or (São Paulo, SP, Brazil) since its inauguration (May 27, 2019, to April 30, 2023). This study was carried out according to the Strengthening the Reporting of Observational Studies in Epidemiology (STROBE) guidelines^38^. All patients signed a consent form (regarding the anesthesia and the endoscopic procedure) prior to the exam after understanding the benefits and possible complications. Approval by the Institutional Review Board was obtained prior to data collection (Ethics Committee ID No.: 556-23-ONCO-VNS-SP-U-I – NAPE - Núcleo de Apoio à Pesquisa e Ensino).

### Patient selection

Only asymptomatic patients at standard risk for CRC (patients over 45 years old) who underwent a screening colonoscopy were included. History of previous colorectal surgery, hereditary colorectal polyposis, and other intestinal syndromes were excluded.

### Technical aspects

#### Bowel preparation

Following institutional protocol, all patients received instructions from nurses regarding bowel preparation. Colon preparation solution includes 500mL of mannitol mixed with 500 mL of Lemon Isotonic drink, and 30 mL (2.250 mg) of simeticone. The solution is recommended 6 hours before the procedure. In addition, a low residual and clear liquid diet is recommended 2 days prior the procedure. The quality of intestinal preparation was assessed using the Boston Bowel Preparation Scale (BBPS), considering=6 an adequate bowel preparation^23^.

#### Pre-procedure care

All patients scheduled for a colonoscopy receive thorough care from the nursing team. This includes admission, checking whether the patient has any person accompanying them who is older than 18 years old, checking all safety measures, basic monitoring (sphygmomanometry, electrocardiography, and pulse oximetry), escorting the patient to the procedure room, verifying and confirming the patient’s identity, and the proposed procedure. Patients also undergo a pre-anesthetic evaluation, which includes assessing the airway, confirming preoperative fasting (at least 8 hours for solid foods, 2 hours for clear liquids, and 4 hours for mannitol)^29^, and reviewing existing medical conditions and medications in use, especially anticoagulants, antiplatelets, and GLP1 receptor agonists. The latter are of particular concern due to the increased risk of residual gastric content in patients not adequately suspended before the examination^36^.

#### Procedure

All procedures were performed under sedation (moderate to deep sedation) assisted by an anesthesiologist, ensuring strict compliance with national regulatory safety standards^7^. Additional oxygen is provided through an oxygen catheter at a flow rate of up to 3 l/min (patients undergoing esophagogastroduodenoscopy and colonoscopy), or a facial mask with a reservoir and a flow rate of 5 l/min (patients undergoing colonoscopy alone). All patients receive eye protection with tape occlusion to prevent corneal abrasion^25^. Fentanyl is commonly administered at a dose of 0.5 to 1 mcg/kg, along with bolus doses of propofol at 1 to 1.5 mg/kg, followed by intermittent boluses of 10 to 20 mg, to maintain the desired anesthetic depth. In some cases, midazolam is used at doses of 2 to 5 mg.

All colonoscopies were performed using an Olympus CF H 190 with EVIS EXERA III CV-190 video system, under CO2 insufflation and water pump machine assistance. The withdrawal time lasted a minimum of 6 minutes. Lesions smaller than 2 cm were promptly resected, while larger lesions were scheduled for subsequent resection following discussions with both patient and attending physician.

#### Post-procedure care

After the procedure, according to the Aldrete scale^1^, patients were only discharged after vital signs assessment, including heart and respiratory rates, level of consciousness, blood pressure, and oxygen saturation. This protocol meticulously evaluates these specific parameters to determine the patient’s suitability for post-anesthesia recovery^6^. Furthermore, before discharge, all patients underwent a reevaluation performed by both the anesthesia team and the endoscopist, providing procedure findings and post-procedure recommendations, including restrictions and possible late adverse events (AEs).

#### Data collection process

Patient data, such as age, gender, and hospital identification (ID) number, were gathered from an electronic spreadsheet (Excel, Microsoft Excel^®^ 2016) that documented all colonoscopies performed at our institution. The Hospital’s data system (TASY Phillips software) was used to assess the outcomes after the colonoscopies, including the adequacy of intestinal preparation and late adverse events.

#### Outcomes and definitions

The primary outcome was the ADR, defined as identification of at least one adenoma during the screening colonoscopy. Secondary outcomes include Polyp Detection Rate (PDR) defined as the percentage of colonoscopies in which at least one polyp is detected and AEs classified based on the recent Adverse Events in Gastrointestinal Endoscopy (AGREE) scale^27^ adapted from The Clavien-Dindo classification for surgical AEs^13^, specifically designed for endoscopic procedures. Post-procedure adverse events were also assessed. The subgroup analysis categorized patients into two groups (45 to 59 and 60 to 75 years old). Additionally, the progression of the ADR and PDR over the years, from the establishment of the hospital to the present day, was checked.

### Statistical analysis

The Pearson’s chi-squared test was employed for categorical variables. Variables with a normal distribution were compared using the independent t-test for two samples. If the assumption of normality was not met, the Mann-Whitney test was employed for group comparisons. Simple linear regression was carried out for the univariate analysis of rates to identify any linear trends. To quantify the effect size of the association between two categorical variables, a logistic regression model was employed to estimate the odds ratio^24,26^. For all analyses, a p-value equal or less than 0.05 was considered statistically significant. The statistical software R, version 4.3.1 (R Foundation for Statistical Computing) was used for all analyses.

## RESULTS

**Figure 1:**
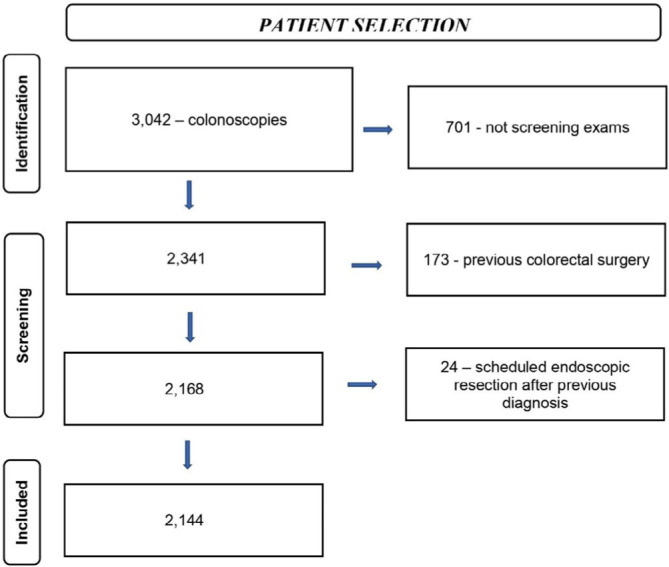
Flow chart — Patient selection.

A total of 3,042 patients undergoing screening colonoscopy were evaluated. From these patients, 898 were excluded as detailed in Figure 1. Thus, a total of 2,144 patients were included in the analyses.

There was a slightly predominance of female patients (52.1%). The mean age was 60.54 years old, with 975 patients aged 45–59 (45.5%) and 1,169 aged 60–75 years (54.5%) (Table 1). The vast majority of patients (99.96%) had adequate bowel preparation, with a mean Boston Scale value of 8.4.

**Table 1:** Demographic data.

Variables	Number of patients (%)
Gender	
Female	1,117 (52.1)
Male	1,027 (47.9)
Age (years old)	
45–59	975 (45.5)
≥60	1,169 (54.5)
Bowel preparation	
Adequate	2,143 (99.96)
Inadequate	1 (0.04)

### Adenoma detection rate

**Figure 2:**
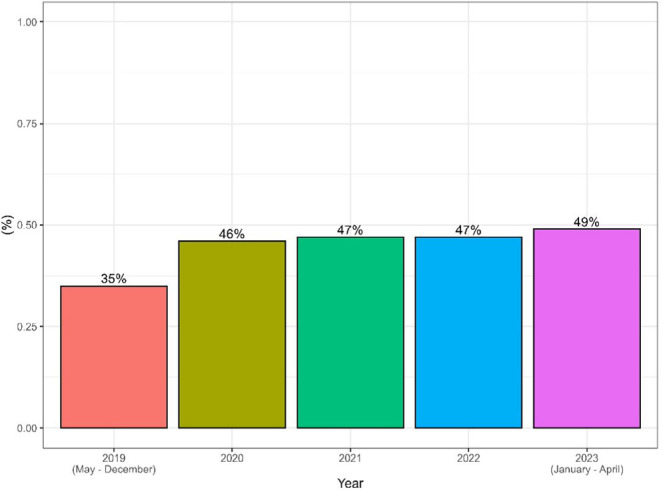
Adenoma detection rate over the years.

From 3,646 removed polyps, 2,071 were adenomas, leading to an ADR of 46.8%, with an average of 2 adenomas per patient (Table 2). The ADR increases over the years (Figure 2).

**Table 2:** Adenoma detection rate.

Variables	Results
Adenoma detection rate*	46.8%
Total number of removed adenomas^†^	2,071
Number of adenomas removed per patient^‡^	2.00 [1.00–3.00]

* Values expressed as %; ^†^Value expressed as n; ^‡^Values expressed as median (percentile 25, 75%).

### Polyps

**Figure 3:**
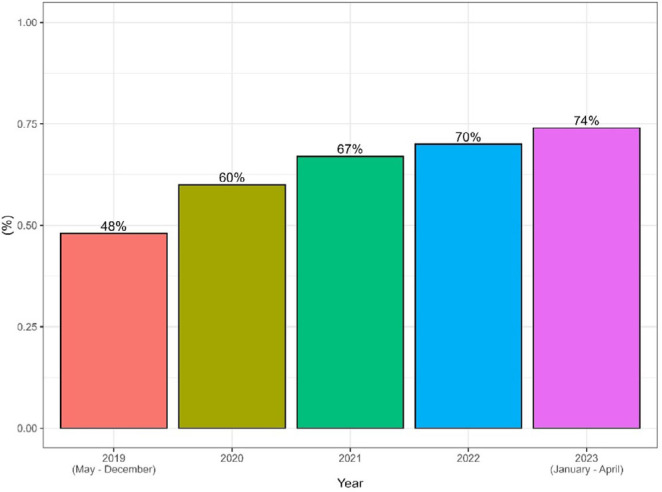
Polyp detection rates over the years.

A total of 3,646 polyps were identified during colonoscopies (68.6%), with a median of 2 polyps per exam. A total of 853 patients had more than one polyp (39.7%) per colonoscopy (Table 3). The PDR increases over the years (Figure 3).

**Table 3:** Polyp detection rate.

Variables	Results
Polyp detection rate*	68.6%
Total number of removed polyps^†^	3,646
Number of polyps removed per patient^‡^	2.00 [1.00–3.00]

* Values expressed as %; ^†^Value expressed as n; ^‡^Values expressed as median (percentile 25, 75%).

### Correlation between age and colonoscopy outcomes

In the univariate analysis, 63.3% of individuals aged between 45–59 years old had at least one polyp compared to 74.9% of those over 60 years old (p<0.001; OR 1,72; 95%CI 1.43–2.07). Regarding ADR, the difference ranged from 35.5% in the 45-59 year-old group versus 58.1% of the group of those over 60 years old (p<0.001; OR 2.23; 95%CI 1.87–2.65).

### Correlation between gender and colonoscopy outcomes

In the univariate analysis, 64.6% of the female group had at least one polyp. Regarding ADR, the female group showed a rate of 42.6% versus 51.4% of the male group (p<0.001; OR 1.42; 95%CI 1.20–1.69).

### Safety profile

A total of 5 AEs (0.23%) were reported, including two late bleedings after polypectomy, both successfully treated in the second colonoscopy with the scope clips (TTSCs) (5^th^ and 7^th^ post-procedure day), one left flank trauma after the patient fell on the floor due to dizziness after deep sedation, which did not require intervention or lead to any sequelae, one perforation during EMR, which was immediately treated with TTSCs, and one aspiration, effectively treated with antibiotics on an outpatient basis. There was no death.

## DISCUSSION

This study provides compelling evidence that achieving outcomes comparable to those seen in most developed countries is possible in developing countries, particularly if adequate conditions are provided. The crucial role for reducing mortality rates emphasizes the importance of maintaining high standards within endoscopy centers. By closely following recommendations from established guidelines^8,30,31,35^, these centers can greatly improve patient outcomes. The solid results of our study has the potential to encourage other centers in our country and also other developing countries to enhance their ADR, which has been considered the most important quality indicator^2^.

Compared to most studies worldwide, our results showed a high ADR (46.8%), exceeding the quality indicator rate recommended by both U.S. Multi-Society Task Force V^30^ and the European Society of Gastrointestinal Endoscopy (ESGE)^4^, both presenting the same ADR of 25% for general screening population. Our results are related to several factors. First, all endoscopists are certified by the Brazilian Society of Endoscopy (SOBED) and have over 5 years of experience across both public and private institutions. Second, the presence of skilled certified anesthesiologists during procedures substantially contributes to raising the quality of all endoscopic procedures, ensuring a safe and more accurate examination. Furthermore, appropriate infrastructure, well-trained nursing technicians, and high quality endoscopic equipment plays an important role to achieving the desired results. In addition, intestinal preparation was adequate in all, except one, patient, which is a determining factor in ADR^30^. This may be related to the use of Mannitol for colon preparation. Despite the fear of several endoscopists around the world regarding colon explosion associated with mannitol-based preparation, various studies have demonstrated that mannitol is as safe as Polyethylene glycol (PEG)^12,15,16^. In our clinical practice, aiming to reinforce procedure safety, electrocautery is only used after cecal intubation and aspiration of the colonic gas. Additionally, the procedure is always performed under CO_2_ insufflation. Although several studies consider mannitol preparation as effective as others when considering the (BBPS) >6 points, in our experience, mannitol use results in higher BBPS points compared to other preparations, justifying its use^15,16^. In Brazil, similar to our group, most centers prefer mannitol to other solutions. Nevertheless, its use is still controversial due its off-label indication as there is no recommendation for oral intake. Simeticone is also important as it reduces bubbles that impair adequate mucosal visualization and may reduce procedure time^10^. Additionally, the guidance given to patients by an extremely competent nursing team and the high educational level of our patients are key for an adequate colon cleansing.

When comparing our results with other Brazilian centers, we noticed that our ADR was considered superior to other observational studies^11,22^. Furthermore, we presented similar results of two randomized controlled trials conducted in Brazil^17,28^. Even though those studies showed a slightly superior ADR, we believe that non-controlled observational studies provide a more reliable picture of the screening colonoscopy status. Despite a lower ADR, our study reported the detection of more adenomas per patient^5^.

Our study revealed a significantly higher PDR (68.6%) and ADR (46.8%) compared to the ESGE recommendation of >25% for general screening population and 30% for males. Our study demonstrated a significantly higher ADR in males (51.4%) compared to females (42.6%). In our practice, we thoroughly examined not only the cecum but also the distal ileum during every colonoscopy. This comprehensive approach allows the diagnosis not only related to inflammatory bowel diseases, such as neoplasms^14^.

We experienced a gradual PDR and ADR increase over the years. The low number of procedures in 2020 is likely related to the COVID-19 pandemic. Despite this challenging scenario, there was no significant reduction in PDR and ADR.

In terms of safety, we experienced a lower AEs rate compared to most studies available in the literature^11,22^. All AEs were solved either through pharmacological treatment or endoscopic intervention. The only case of aspiration was related to the use of semaglutide. The use of drugs analogous to GLP-1 has been increasing and has become an issue for endoscopists, surgeons, and anesthesiologists^36^. To ensure patients safety, our institution updated our protocol, requiring patients to discontinue its use 21 days before the procedure^36^.

This study is not exempt from limitations. The retrospective nature of this study inherently carries limitations. Hypothesis testing and the exclusion of potential confounders could not be carried out. Nevertheless, we believe that a non-controlled study reflects the real scenario of screening colonoscopies. Although this study did not present the withdraw time, our service adheres to a long scheduling interval (1 hour per patient) providing time for a careful evaluation.

Despite these limitations, our results promote a better understanding of several measures to enhance the quality of screening colonoscopy, potentially motivating other services to improve ADRs.

## CONCLUSIONS

This study demonstrated that high quality screening colonoscopies can be performed in developing countries, especially when performed by experienced endoscopists and trained nurses, under an adequate infrastructure.
